# Prevalence, diagnosis and management of intracranial atherosclerosis in White populations: a narrative review

**DOI:** 10.1186/s42466-024-00341-4

**Published:** 2024-11-11

**Authors:** Evangelos Panagiotopoulos, Maria-Ioanna Stefanou, George Magoufis, Apostolos Safouris, Odysseas Kargiotis, Klearchos Psychogios, Sofia Vassilopoulou, Aikaterini Theodorou, Maria Chondrogianni, Eleni Bakola, Frantzeska Frantzeskaki, Tatiana Sidiropoulou, Stavros Spiliopoulos, Georgios Tsivgoulis

**Affiliations:** 1 Second Department of Neurology, “Attikon” University Hospital, School of Medicine, National and Kapodistrian University of Athens, Athens, Greece; 2 Interventional Neuroradiology Unit, Metropolitan Hospital, Piraeus, Greece; 3 Stroke Unit, Metropolitan Hospital, Piraeus, Greece; 4 First Department of Neurology, School of Medicine, Eginition Hospital, National and Kapodistrian University of Athens, Athens, Greece; 5 Second Department of Critical Care, ‘Attikon’ University Hospital, School of Medicine, National and Kapodistrian University of Athens, Athens, Greece; 6 Second Department of Anesthesiology, “Attikon” University Hospital, School of Medicine, National and Kapodistrian University of Athens, Athens, Greece; 7 Interventional Radiology Unit, Second Department of Radiology, ’Attikon’ University General Hospital, Athens, Greece

**Keywords:** Intracranial atherosclerosis, Stroke, Endovascular, Stenting, Intracranial artery stenosis

## Abstract

**Background:**

Intracranial atherosclerotic disease (ICAD) represents a leading cause of ischemic stroke worldwide, conferring increased risk of recurrent stroke and poor clinical outcomes among stroke survivors. Emerging evidence indicates a paradigm shift, pointing towards increasing detection rates of ICAD among White populations and an evolving epidemiological profile across racial and ethnic groups. The present review aims to provide a comprehensive overview of ICAD, focusing on its pathophysiology, diagnostic approach, and evolving epidemiological trends, including underlying mechanisms, advanced neuroimaging techniques for diagnostic evaluation, racial disparities in prevalence, and current and emerging management strategies.

**Main body:**

Atherosclerotic plaque accumulation and progressive arterial stenosis of major intracranial arteries comprise the pathophysiological hallmark of ICAD. In clinical practice, the diagnosis of intracranial artery stenosis (ICAS) or high-grade ICAS is reached when luminal narrowing exceeds 50% and 70%, respectively. Advanced neuroimaging, including high-resolution vessel wall MRI (HRVW-MRI), has recently enabled ICAD detection before luminal stenosis occurs. While earlier studies disclosed significant racial disparities in ICAS prevalence, with higher rates among Asians, Hispanics, and Blacks, recent evidence reveals rising detection rates of ICAD among White populations. Genetic, environmental and epigenetic factors have been suggested to confer an increased susceptibility of certain ethnicities and races to ICAD. Nevertheless, with improved accessibility to advanced neuroimaging, ICAD is increasingly recognized as an underlying stroke etiology among White patients presenting with acute ischemic stroke and stroke of undetermined etiology. While conventional management of ICAS entails risk factor modification, pharmacotherapy, and endovascular treatment in selected high-risk patients, substantial progress remains to be made in the management of ICAD at its early, pre-stenotic stages.

**Conclusion:**

ICAD remains a critical yet underappreciated risk factor for ischemic stroke across all populations, highlighting the need for increased awareness and improved diagnostic strategies. The emerging epidemiological profile of ICAD across racial groups necessitates a reassessment of risk factors, screening protocols and preventive strategies. Future research should focus on refining the diagnostic criteria and expanding the therapeutic options to cover the full spectrum of ICAD, with the aim of improving patient outcomes and reducing the global burden of intracranial atherosclerosis and stroke.

## Background

Intracranial atherosclerotic disease (ICAD) represents a leading cause of ischemic stroke worldwide that is associated with a high recurrence risk and poor clinical outcomes among stroke survivors [1]. With the global incidence and lifetime risk of ischemic stroke rising, ICAD remains currently underrecognized [2]. ICAD constitutes a facet of the atherosclerotic continuum that unfolds within the cerebral arteries and correlates with established pro-atherosclerotic risk factors, including hypertension, diabetes, smoking, dyslipidemia and metabolic syndrome [3]. Progressive accumulation of atherosclerotic plaque entails down- and up-stream pro- and anti-inflammatory cascades, that elicit vascular changes ranging from arterial-wall thickening to progressive luminal stenosis, with the latter defined as intracranial artery stenosis (ICAS) [4]. Although atherosclerosis is conceptualized as a continuum within the cerebral vasculature, in clinical practice, the terms ICAD and ICAS are reserved for the atherosclerotic processes within the large and medium-sized intracranial arteries [5].

Until recently, it was widely accepted that significant racial and ethnic disparities exist in the prevalence of ICAD and ICAS, yet recent evidence has challenged this notion [6]. Previous epidemiological studies have identified ICAS as underlying cause of acute ischemic stroke (AIS) in 30 to 70% of Asian, Hispanic, and Black patients, compared to a markedly lower prevalence of 5 to 10% in White patient populations [7]. In addition, genetic, environmental, and epigenetic factors have been suggested to confer an increased susceptibility of certain ethnicities and races to ICAD [5, 8–10]. Nevertheless, recent research has indicated a paradigm shift, with evidence pointing towards increasing detection rates of ICAD among White patients presenting with AIS or stroke of undetermined etiology [6]. These findings suggest a more uniform distribution of ICAD across ethnic and racial groups than previously thought, arguing that ICAD may comprise an underrecognized cause of cryptogenic stroke in clinical practice. In light of the emerging data in the literature, the aim of the present review is to summarize the state of current evidence concerning the latest epidemiological data on racial and ethnic disparities, as well as the diagnostic and therapeutic approaches of ICAD. Additionally, the present review aims to highlight potential knowledge gaps and discuss future research directions.

## Pathophysiology of ICAD

**Fig. 1:**
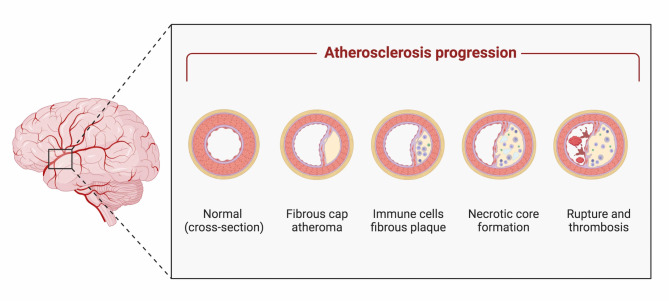
Pathophysiology of Intracranial Atherosclerosis. Intracranial atherosclerotic disease (ICAD) is a chronic inflammatory disorder affecting arterial segments with high endothelial shear stress, such as bifurcations and curvatures. Early in atherogenesis, oxidized low-density lipoproteins (LDL) accumulate in the arterial intima, activating endothelial and smooth muscle cells (SMCs). This prompts monocytes to become macrophages and dendritic cells, which then differentiate into foam cells. As atherosclerosis progresses, immune cell infiltrates contribute to the thinning of the fibrous cap. Lipid accumulation and foam cell necrosis eventually form a necrotic core. At advanced stages of atherosclerosis, neovascularization from the adventitial vasa vasorum may lead to intraplaque hemorrhage and plaque instability. Image created with Biorender

ICAD is a chronic inflammatory disorder that predominantly arises in arterial segments subjected to high endothelial shear stress, such as points of arterial bifurcations and inner curvatures [11]. Cardiovascular risk factors, including hypertension, dyslipidemia, smoking, obesity, and diabetes, are known to induce the inflammatory process, increasing the endothelial permeability particularly in regions that are prone to vascular injury [7]. Subsequent to the initial stages of atherogenesis, low-density lipoproteins (LDL) accumulate within the arterial intima and undergo oxidation, thereby activating both endothelial and smooth muscle cells (SMCs) and prompting monocytes to migrate and differentiate into macrophages and dendritic cells [11]. These cells, harboring lipid deposits from oxidized LDL, secrete pro-inflammatory cytokines and eventually differentiate into foam cells. Progressively, as atherosclerosis advances, accumulation of lipids leads to the expansion of the lipid core within the intima. At the advanced stages of atherosclerosis, the necrosis of both foam cells and SMCs leads to the formation of a necrotic core. Concurrently, neovascularization from the adventitial vasa vasorum may result in intraplaque hemorrhage and plaque instability (Fig. 1).

**Fig. 2:**
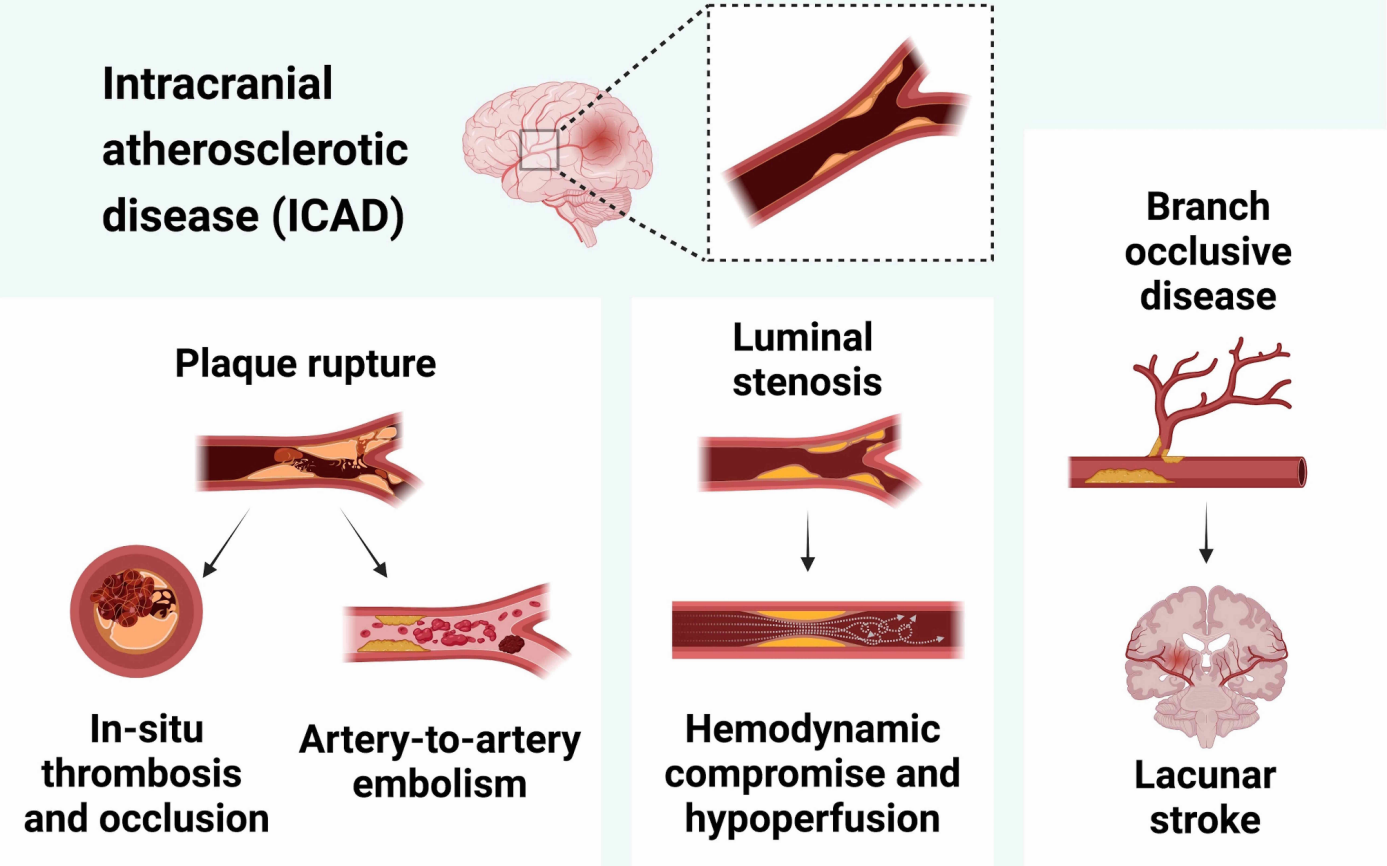
Mechanisms of ischemic stroke in Intracranial Atherosclerosis. Intracranial atherosclerotic disease (ICAD) can lead to ischemic events through various pathophysiological mechanisms, either individually or in combination. These comprise: (i) in-situ thrombosis and occlusion of the parent artery; (ii) artery-to-artery embolism, both resulting from atherosclerotic plaque rupture; (iii) progressive luminal stenosis causing hemodynamic compromise and cerebral hypoperfusion; and (iv) branch occlusive disease due to plaque expansion over small perforator artery openings, that leads to subcortical and/or lacunar strokes in the presence of ICAD. Adapted by Chen et al. [18] Image created with Biorender

ICAD can coexist with systemic atherosclerosis affecting different arterial beds, including extracranial, coronary, or peripheral arteries, or it may manifest in isolation. Regarding localization, the prevalence of ICAD/ICAS is higher in the anterior circulation compared to the posterior circulation [12–15]. More specifically, the prevalence of intracranial artery stenosis has been reported to be twofold higher in the anterior compared to the posterior circulation (approximately 60% vs. 30% among patients with ICAS) [12–15]. Contributing to the instability and inflammatory susceptibility of plaques, intracranial arteries lack an external elastic lamina, often exhibit impaired complement regulation, and are characterized by the presence of ubiquitin-proteasome complexes and activated nuclear factor κB [16, 17]. ICAD may cause ischemic events through various pathophysiological mechanisms that may occur individually or in combination [7, 18]. These comprise: (i) in-situ thrombosis and occlusion of parent artery; (ii) artery-to-artery embolism, (iii) progressive luminal stenosis that precipitates cerebral hypoperfusion, or (iv) branch occlusive disease due to plaque expansion covering small perforator artery openings (Fig. 2).

### Current definitions and prevalence of ICAD and ICAS

Epidemiological data on ICAD and ICAS are highly contingent upon their definitions and the diagnostic modalities used for their identification. Prior studies have primarily focused on ICAS prevalence among symptomatic patients, with AIS or transient ischemic attack (TIA), utilizing conventional catheter angiography, computed tomography (CT) angiography, and transcranial Doppler ultrasound (TCD) [19, 20]. Yet, the widespread implementation of advanced neuroimaging techniques has recently facilitated ICAD diagnosis in both symptomatic and asymptomatic individuals. These include high-resolution magnetic resonance imaging (HR-MRI), Magnetic Resonance Angiography (HR-MRA), and vessel wall MRI (HRVW-MRI), which enable the early detection of atherosclerotic plaque on the vessel wall, indicative of ICAD, before luminal stenosis (i.e., ICAS) occurs.

According to current definitions, ICAD is defined as a progressive pathological process characterized by atherosclerotic plaque formation affecting major intracranial arteries at any stage of the disease [4, 7, 21]. Any evidence on pathological or neuroimaging studies of stenotic or non-stenotic plaque in large or medium-sized intracranial arteries qualifies as ICAD [22]. ICAS is diagnosed when plaque accumulation narrows the arterial lumen by 30 to 99%. However, as per current clinical practice guidelines, ICAS typically refers to a luminal narrowing that exceeds 50% as detected by angiographic techniques or TCD [21]. High-grade ICAS, accordingly, refers to arterial caliber stenosis exceeding 70% and may be associated with hemodynamic compromise in the downstream arterial territory [21].

A standardized method for ICAS quantification on conventional angiography was first established by the Warfarin-Aspirin Symptomatic Intracranial Disease (WASID) trial [12]. Based on WASID measurement criteria, percent stenosis was defined as [(1 − (Dstenosis/Dnormal))] × 100, where Dstenosis was defined as the diameter of the artery at the site of the most severe stenosis and Dnormal as the diameter of the proximal normal artery [23]. If the proximal segment was diseased, contingency sites were chosen to measure Dnormal from distal arteries (second choice) or feeding arteries (third choice). This method showed high rates of inter-observer and intra-observer agreement for major intracranial arteries. Subsequently, standardized diagnostic criteria for ICAS quantification have been developed and clinically validated, that mainly rely on the assessment of cerebral blood flow dynamics and lumen caliber, utilizing TCD, CT or MR angiography [24, 25].

Regarding the prevalence of ICAS and ICAD, the advancements witnessed in neuroimaging over the past few years have shifted the focus from ICAS to ICAD diagnosis and from symptomatic to asymptomatic populations. Previous studies have indicated a higher prevalence of ICAS in Asian, Hispanic, and Black patients compared to White patient populations [7, 13, 26]. ICAS was detected in < 10% of Whites presenting with AIS compared to over 50-60% in Asians. Hispanic and Black patients have also been found to harbor an almost five-fold increased risk of ICAS-related strokes compared to Whites [10, 27]. The fact that ICAS is more prevalent among Asians, Hispanics and Blacks compared to White populations has been reported to date in several studies using diverse ICAD/ICAS definitions and diagnostic modalities. Nevertheless, recent evidence has indicated that the prevalence of ICAD among Whites may have been underestimated [28].

Observational studies evaluating the prevalence of intracranial artery calcifications as ICAD marker using novel neuroimaging techniques in symptomatic White patients (i.e., presenting with AIS or TIA) have yielded ICAD estimates of over 50%; rates that align with those previously reported in non-White populations [6, 29].

Recent studies have also demonstrated that over half of patients with stroke of undetermined etiology present with underlying ICAD [6]. In particular, a recently published cohort analyzed data from the Erasmus Stroke Study (ESS) registry, which included 943 patients (561 with ischemic stroke and 382 with TIA) who underwent CT-angiography using a standardized optimized contrast-enhanced protocol. In this study, the prevalence of intracranial artery calcification as ICAD marker was estimated at 58.5% among patients with stroke of undetermined etiology. Importantly, in patients with embolic stroke of undetermined source (ESUS), an elegant study by Ameriso et al. documented a prevalence of ICAD of 16% among ESUS patients, capitalizing on the well-defined dataset of the NAVIGATE ESUS trial [30]. Concerning the laterality of ICAD, several studies have documented a significantly higher prevalence of ICAD ipsilateral to ischemic stroke compared to contralateral (64% vs. 43%; odds ratio [OR]: 5.25; 95% CI: 2.83 to 9.73) [31, 32]. This reinforces the hypothesis that ICAD may comprise an undetected mechanism implicated in the pathophysiology of otherwise cryptogenic stroke [33].

Regarding asymptomatic ICAS, prevalence rates of up to 12% have been reported in the general population, with ≥ 50% and ≥ 70% ICAS being reported in approximately 9% and 4%, respectively [19, 34, 35]. Even more interesting are the recent findings of population-based studies assessing the prevalence of ICAD in asymptomatic White individuals, reporting prevalence estimates of ICAD exceeding 80% in community cohorts using as surrogate marker the presence of intracranial artery calcifications [36].

In summary, the prevalence of symptomatic ICAS in White populations is approximately 10%, while asymptomatic ICAD might be found in up to 50% of patients. These data underscore the need for a more comprehensive screening for underlying ICAD, particularly in symptomatic patients presenting with stroke of undetermined etiology.

### Inter-racial differences in ICAD and ICAS

Taking the observed inter-racial differences in ICAD and ICAS prevalence into account, several issues should be considered. First, it is still widely accepted that ICAD is less prevalent in Whites than non-Whites, while the opposite holds true for extracranial atherosclerosis [37]. Although the reasons behind this discrepancy remain to date only partially elucidated, the higher prevalence of hypertension among Asians and Blacks has been associated with a higher predisposition to atherosclerosis of intracranial arteries [37]. Second, inter-racial differences in factors like the vascular tortuosity and elasticity of intracranial arteries, have also been reported and linked to genetic differences in elastin metabolism, that predispose to arterial wall shear-stress [37]. Third, additional genetic polymorphisms, including the RNF213 (ring finger protein 213) genetic variant that has been linked to vascular fragility, along with genetic variants, including α-adducin, angiotensinogen, and aldosterone synthase, may account for a higher risk of ICAD in East Asian populations, but lower in Whites [19].

Notwithstanding the previous considerations, recent evidence on the exponential rise of ICAD among Whites suggests evolving prevalence rates across racial and ethnic groups. First, the rise in cardiovascular risk factors, including dietary salt-induced hypertension, is becoming more prevalent among Whites due to lifestyle changes [4, 5, 38]. Second, environmental and epigenetic factors might be diminishing the previously noted racial disparities in the incidence of ICAD [39]. Third, the increasing global awareness of ICAD risk factors and the standardized stroke prevention strategies appear to be reducing these disparities. Fourth, it is crucial to note that previous epidemiological studies provided increased prevalence estimates of ICAD in non-Whites, and particularly East Asian populations. However, these epidemiological studies likely suffered from overreporting, as recent research using HRVW-MRI has indicated that Moyamoya disease and vasculitis, which often represent the underlying etiology of ICAS in Asians, may have been misclassified as ICAD in earlier studies [37].

### Diagnostic approach of ICAD and ICAS

**Fig. 3:**
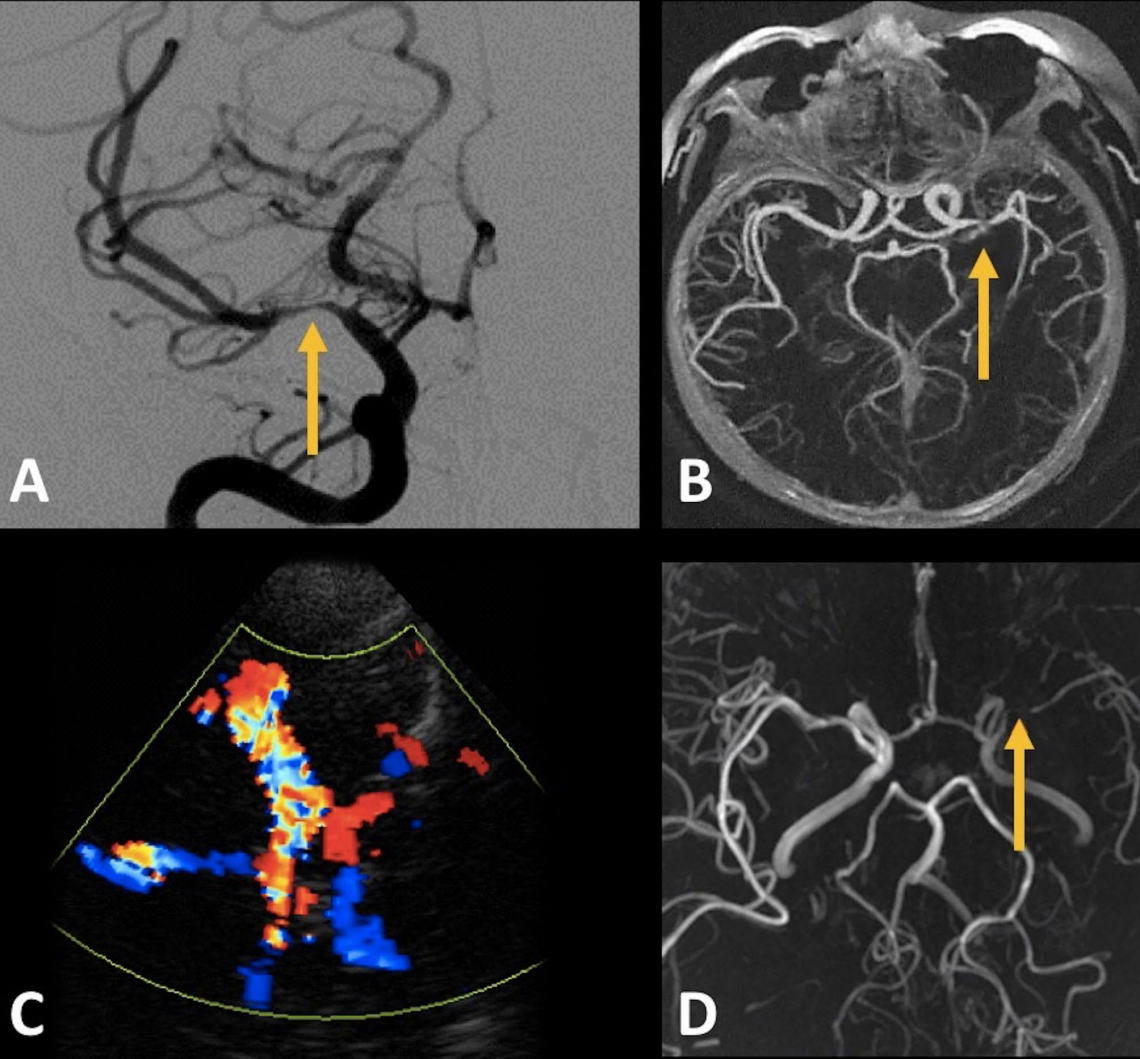
Diagnostic modalities for Intracranial Atherosclerosis. **A**: DSA reveals atherosclerotic stenosis of the M2 segment of the right MCA; **B**: Axial CTA shows high-grade stenosis of the M1 segment of the left MCA; **C**: Transcranial color-coded duplex ultrasonography (TCCS) reveals significant stenosis of the M1 segment of the right MCA with aliasing phenomenon; **D**: Axial MRA shows a high-grade stenosis of the M1 segment of the left MCA Abbreviations: DSA: Digital Subtraction Angiography; MCA: middle cerebral artery; CTA: Computed Tomography Angiography; MRA: Magnetic Resonance Angiography

The increasing recognition of the causal association between AIS and ICAD has shifted the clinical focus from ICAS to the early, non-stenotic stages of ICAD. Novel imaging modalities, including HRVW-MRI, and improved MRI protocols and sequences, not only enhance our ability to quantify luminal caliber but also provide deeper insights into the vessel wall pathology [40]. These techniques, combined with traditional modalities, including TCD, yield comprehensive measurements of luminal narrowing and hemodynamic changes, often reaching or surpassing the diagnostic accuracy of Digital Subtraction Angiography (DSA), the traditional gold standard (Fig. 3) [40].

DSA has historically served as the gold-standard modality for ICAS diagnosis [19]. With its high-resolution and visualization capabilities for the intracranial vasculature, DSA enables the quantification of the degree of intracranial stenosis and the estimation of the collateral supply, but also drives the differential diagnosis by ruling out alternative causes of ICAS such as Moyamoya disease or vasculitis [19]. However, DSA’s inability to depict the arterial wall does not allow detection of early non-stenotic ICAD. Additionally, its invasive nature and associated risks have curtailed its use as a first-line diagnostic tool [19]. By contrast, noninvasive angiography methods, encompassing CT angiography (CTA) and MRA offer the advantages of safety, speed, and accessibility; yet with variable sensitivity and specificity for ICAS/ICAD detection, especially of the medium sized vessels (Table 1) [19]. Alongside neuroimaging, TCD serves as a noninvasive bedside tool for assessment of ICAS and cerebral blood flow hemodynamics [41, 42].

**Table 1:** Diagnostic modalities used for clinical assessment of intracranial atherosclerosis [4, 7, 87]

Diagnostic Modality	Indications/ Benefits	Contraindications/ Limitations	Sensitivity Compared to DSA	Specificity Compared to DSA	Positive Predictive Value Compared to DSA	Negative Predictive Value Compared to DSA
CTA	1. Widely available 2. Rapid 3. Non-invasive	1. Radiation 2. Contrast risks 3. Artifacts due to calcifications or susceptibility gradients	~ 97% for 50%-99% stenosis	~ 99% for 50%-99% stenosis	~ 93% for 50%-99% stenosis	~ 99% for 50%-99% stenosis
TOF-MRA	1. Non-invasive 2. No radiation	1. Blood-flow artifact 2. MRI contraindications 3. Claustrophobia 4. Lengthy procedure	78-85% for 50-99% stenosis	~ 95% for 50-99% stenosis	75-79% for 50-99% stenosis	95-97% for 50-99% stenosis
Contrast-enhanced MRA	1. Non-invasive 2. No radiation 3. No blood-flow artifacts	1. Contrast risks 2. MRI contraindications 3. Claustrophobia 4. Lengthy procedure	~ 66% for 50%-69% stenosis ~ 95% for 70%-99% stenosis	~ 94% for 50%-69% stenosis ~ 92% for 70%-99% stenosis		
HRVW-MRI	1. Depiction of non-stenotic atheromatic plaques/ intraplaque elements/unstable plaques 2. Characterization of stenosis etiology 3. Non-invasive 4. No radiation	1. Costly 2. MRI contraindications 3. Claustrophobia 4. Lengthy procedure	~ 69.2% for 50%-99% stenosis ~ 80% for 50%-99% stenosis or occlusion	~ 53.6% for 50%-99% stenosis ~ 53.6% for 50%-99% stenosis or occlusion		
DSA	1. Gold standard 2. Detailed vascular visualization 3. Collateral circulation depiction 4. Differential diagnosis (i.e., exclusion of non-atherosclerotic ICAS) 5. Can be combined with treatment in same session	1. Invasive 2. Low but existent stroke risk 3. Costly 4. Radiation 5. Contrast risks (i.e., increased risks in renal failure) 6. Hemorrhagic risk				
TCD	(1) Non-invasive (2) Rapid 3. No contrast or radiation 4. Microemboli detection 5. Assessment of vasomotor reactivity 6. Dynamic monitoring 7. Bedside test	1. Operator dependent 2. Technical limitations 3. No visualization of the vessel wall	90-99% for > 50% stenosis or occlusion of MCA 70-80% for > 50% stenosis or occlusion of Intracranial segment of vertebral and basilar artery	90-99% for > 50% stenosis or occlusion of MCA 90-99% for > 50% stenosis or occlusion of Intracranial segment of vertebral and basilar artery	~ 36%	~ 86%

Abbreviations:

CTA: Computed tomography angiography; DSA: Digital subtraction angiography; HRVW-MRI: High resolution vessel wall magnetic resonance imaging; ICAS: Intracranial atherosclerotic stenosis; MCA: Middle cerebral artery; MRI: Magnetic resonance imaging; TCD: Transcranial doppler ultrasound; TOF-MRA: Time-of-flight magnetic resonance angiography

In clinical practice, TCD provides essential hemodynamic information that static images cannot, including flow direction, collateral circulation, and the detection of microembolic signals (MES) (Table 1). In the Stroke Outcomes and Neuroimaging of Intracranial Atherosclerosis (SONIA) trial, the accuracy of TCD in detecting 50–99% ICAS was compared with DSA, yielding negative and positive predictive values of 86% and 36%, respectively [24]. TCD is additionally used for MES detection in symptomatic and asymptomatic patients with ICAD, serving both as a diagnostic and prognostic tool for evaluating the risk of stroke and stroke recurrence [41]. Moreover, TCD can evaluate vasomotor reactivity (VMR) as an indirect measure of dynamic cerebral autoregulation and collateral circulation at rest or during induced hypercapnia. Impaired VMR in ICAS has been associated with an increased risk of stroke and worse clinical outcomes [41]. Transcranial color-coded duplex sonography (TCCS) and contrast-enhanced TCCS (CE-TCCS) are used to enhance the accuracy of diagnosing ICAS and ICAD by integrating anatomical details of the arterial lumen, while CE-TCCS using the power mode may improve the visualization of intra- and post-stenotic flow [43].

Regarding MR-based angiography, Time-of-Flight MR Angiography (TOF-MRA) and contrast-enhanced MRA are clinically indispensable for the evaluation of the intracranial vasculature (Table 1). TOF-MRA relies on flow-related contrast enhancement [44]. Despite its tendency to overestimate ICAS especially in cases with decreased distal flow, TOF-MRA provides useful hemodynamic data and importantly, without the need of contrast injection [45]. Conversely, contrast-enhanced MRA offers improved morphological visualization, particularly for high-degree stenosis with reduced flow [44]. Quantitative MRA, a relatively recent development, utilizes a phase-contrast technique combined with TOF-MRA to precisely quantify intra-arterial blood flow [46].

CTA surpasses both TCD and MRA in diagnostic accuracy (Table 1) [47]. CTA’s independence from flow dynamics permits higher accuracy in estimating the degree of stenosis of the intracranial vessels. Notably, CTA enables the construction of three-dimensional (3D) models of the intracranial vasculature for blood-flow simulations using computational fluid dynamics (CFD) techniques [48] However, CTA exhibits several limitations, including the inability to visualize the intracranial internal carotid artery near the sphenoid sinus due to susceptibility gradients. Additionally, calcifications of the arterial wall may cause artifacts, limiting its utility for the evaluation of carotid siphon stenosis [7]. These limitations can be partially reduced by employing newer imaging methods, such as Photon-counting detector CT (PCD-CT), which possesses higher spatial and contrast resolution of soft tissues compared to conventional CT, and Dual-Energy CTA (DECTA), which enables bone removal based on three-material (iodine, calcium, and hemorrhage) differentiation and consequently reduces artifacts [49, 50].

Imaging of the brain parenchyma can also provide critical insights into ICAD/ICAS. In this regard, CT perfusion and perfusion-weighted MRI are instrumental in identifying cerebral areas with insufficient blood supply. Brain CT contributes to the identification of arterial calcification, that comprises a sensitive imaging marker of ICAD [36]. Based on the underlying pathophysiology of stroke, specific imaging patterns on brain CT or MRI indicative of ICAS may emerge. In cases of parent artery in-situ thrombosis, localized infarctions within the territorial domain of supplying arteries are commonly observed [51]. In addition, artery-to-artery embolism may manifest as one or multiple patchy or wedge-shaped infarcts localized to areas supplied by a specific artery [51]. Finally, hypoperfusion typically results in patchy or wedge-shaped infarcts predominantly within border zone (watershed) regions [51]. Crucially, brain CT or MRI scans should be cautiously evaluated for the presence of clinically silent cerebral infarcts, especially in asymptomatic individuals incidentally diagnosed with ICAD.

In clinical practice, diagnosing non-stenotic ICAD poses significant challenges that necessitate a multimodal approach, extending beyond traditional imaging techniques primarily focused on ICAS. HRVW-MRI has emerged as an indispensable tool in this regard, providing detailed characterization of both luminal morphology and vascular wall (Table 1). This modality is adept at identifying both non-stenotic and unstable atheromatous plaques, offering insights into critical aspects such as fibrous cap thickness, intraplaque hemorrhage, and lipid core dimensions [52]. Most importantly, HRVW-MRI aids in the etiological differentiation of intracranial stenoses, differentiating whether they are due to atherosclerosis or other pathogenic mechanisms such as vasculitis or Moyamoya disease [52].

When evaluating patients with suspected ICAD, it is crucial to consider alternative diagnoses such as vasculitis, dissection, and unilateral arteriopathy of the young [5, 52, 53]. Vasculitis often presents with concentric vessel wall thickening and enhancement on MRI, sometimes with “skip” lesions. Dissections are identified by an intimal flap, double lumen, or crescent-shaped hyperintensity, visible on HRVW-MRI and CTA. Unilateral arteriopathy of the young is characterized by segmental narrowing or occlusion, typically without plaque or wall enhancement, observable on MRI/MRA, CTA, and DSA. Differentiating these conditions through advanced neuroimaging techniques is vital for accurate diagnosis and treatment [5, 52, 53]. In addition, advanced neuroimaging modalities like 3D high-resolution black blood MRA can further contribute to visualizing the vascular wall and enable ICAD diagnosis at its early non-stenotic stages [54].

To summarize, the evolving understanding of the relationship between AIS and ICAD has shifted the clinical focus from ICAS to the early, non-stenotic stages of ICAD, particularly in patients presenting with cryptogenic stroke or ESUS. Advanced imaging techniques such as HRVW-MRI and enhanced MRI protocols provide enhanced capabilities for assessing both vessel lumen and vessel wall pathology. These methods, alongside traditional approaches like TCD, offer comprehensive evaluations of luminal narrowing and hemodynamics, matching the accuracy of DSA.

### Laboratory biomarkers

While laboratory findings alone are not specific for diagnosing ICAD or ICAS, they play a critical role in assessing the overall atherosclerotic risk profile. Elevated levels of white blood cells, neutrophils, and the neutrophil-to-lymphocyte ratio (NLR), along with increased homocysteine levels and decreased lymphocyte counts, have been associated with the presence of ICAD [55]. Additionally, certain lipid ratios such as total cholesterol/high-density lipoprotein-cholesterol (TC/HDL-C), low-density lipoprotein-cholesterol/high-density lipoprotein-cholesterol (LDL-C/HDL-C), remnant cholesterol/high-density lipoprotein-cholesterol (RC/HDL-C), non-HDL-C/HDL-C, apolipoprotein B (apo B)/HDL-C, and apo B/apolipoprotein A-I (apo A-I) have shown diagnostic potential when elevated [55]. Lipoprotein (a) (Lp(a)), especially at levels exceeding 50 mg/dL, also correlates with the presence of ICAD [55]. Moreover, inflammatory biomarkers such as interleukin-6 (IL-6), components of the endothelial glycocalyx, and extracellular matrix metalloproteinases (MMPs) are increasingly recognized for their relevance in atherosclerotic plaque formation [7, 55]. Notably, changes in the endothelial glycocalyx, particularly early reductions in thickness, serve as critical indicators of atherosclerotic plaque development and ICAD manifestation, complementing conventional markers like C-reactive protein (CRP) and high-sensitivity CRP (hsCRP) [56].

### Diagnostic challenges

Despite the diagnostic advancements highlighted above, the etiology of many ischemic events often remains elusive, leading to classifications such as cryptogenic or ESUS [57]. Recent evidence suggests that a significant proportion of these cryptogenic and ESUS strokes are linked to unstable, non-stenotic atherosclerotic plaques that conventional angiographic techniques fail to detect [32, 58, 59]. Differentiating between cryptogenic and ESUS strokes is particularly challenging when thromboembolism originates from intracranial atherosclerotic plaques [59]. Cryptogenic stroke refers to strokes without evident underlying cause; in clinical practice, however, the term also applies to strokes with multiple etiologies or cases with incomplete diagnostic work-up. Conversely, ESUS is more precisely defined to encompass non-lacunar brain infarcts without proximal arterial stenosis or cardioembolic sources [60]. The term was introduced in 2014 with the aims of (i) standardizing a work-up framework and (ii) allowing assessment in the context of randomized-controlled clinical trials to determine if oral anticoagulation versus antiplatelet therapy may decrease the risk of recurrence in ESUS. Although so-far published randomized controlled trials (RCTs) have yielded neutral results, the distinction of ESUS has practical implications, such as (i) indicating targeted investigations into potential embolic sources like patent foramen ovale (PFO), covert atrial fibrillation (AF), and complex aortic arch atheroma; (ii) considering PFO closure in patients with ESUS < 60 years; and (iii) considering empirical anticoagulation or enrollment in ongoing RCTs examining the use of novel oral anticoagulants (NOACs) in selected ESUS patients. Notably, since the inclusion of ICAD patients in the ESUS population may have contributed to the neutral results of previous RCTs comparing NOAC and aspirin in ESUS, future RCTs should consider selecting ESUS patients based on the presence of ICAD [60].

The traditional Trial of Org 10,172 in Acute Stroke Treatment (TOAST) classification, as well as the subsequent Atherosclerosis, Small-vessel disease, Cardiac pathology, Other causes, or Dissection (ASCOD) classification, do not adequately identify non-stenotic atherosclerotic plaques as the primary source of thromboembolism leading to ischemic events [32]. In contrast, the more recent Causative Classification of Stroke (CCS) system has improved the categorization of these events by recognizing non-stenotic atherosclerotic plaques as potential embolic sources, facilitating a more precise classification of ischemic strokes [32]. It is important to note that in patients presenting with cryptogenic stroke or ESUS, comprehensive evaluation, incorporating HRVW-MRI, is recommended when other stroke etiologies have been ruled out, to uncover intracranial atherosclerotic changes that conventional imaging might miss, especially for patients with suspicion of branch atherosclerotic disease related infarcts [61].

### Management of ICAD and ICAS

To date, the majority of RCTs have focused on the management of symptomatic ICAS. By contrast, there is a dearth of evidence from RCTs on symptomatic or asymptomatic patients presenting with atherosclerotic intracranial stenoses less than 50% (i.e., ICAD).

### Medical management

Antithrombotic therapy comprises the cornerstone of pharmacological treatment for patients diagnosed with ICAS. The WASID trial disclosed no significant difference in the rates of stroke recurrence between patients administered warfarin and those treated with aspirin for ICAS ranging from 50 to 99% over a two-year period (Table 2) [12]. Yet, aspirin presented a superior safety profile compared to warfarin, and was associated with significantly lower rates of mortality and major hemorrhagic complications [12]. Following the SAMMPRIS (Stenting and Aggressive Medical Therapy for Preventing Recurrent Stroke in Intracranial Stenosis) trial, dual antiplatelet therapy, comprising aspirin (325 mg) and clopidogrel (loading dose of 300 mg followed by 75 mg on a daily basis) for 90 days, became the standard of care for symptomatic ICAS [62]. The optimal duration of dual antiplatelet therapy for secondary stroke prevention in symptomatic ICAS remains uncertain, with previous RCTs investigating treatment durations of up to 6 months. Weighting the risk of stroke recurrence against the risk of hemorrhagic complications, current American Heart Association/American Stroke Association (AHA/ASA) guidelines recommend dual antiplatelet therapy for 90 days followed by single anti-platelet agent, for patients with recent stroke or TIA attributed to severe (70–99%) ICAS [21, 63]. For patients with a history of stroke or TIA caused by 50–69% ICAS, the AHA/ASA guidelines comment that there is little evidence supporting the benefit of a longer duration of dual antiplatelet therapy compared to that indicated for other causes of stroke. By contrast, the European Stroke Organization (ESO) guidelines recommend dual antiplatelet therapy for up to 90 days after the event, followed by single antiplatelet therapy [21, 63]. Notably, the currently ongoing, 3-arm, double-blind phase III CAPTIVA trial (ClinicalTrials.gov Identifier: NCT05047172), which will randomize patients with high-grade (70–99%) ICAS and recent stroke to 1 year of combined treatment with aspirin (81 mg daily) and: (1) ticagrelor (90 mg twice daily), (2) low-dose rivaroxaban (2.5 mg twice daily), or (3) clopidogrel (75 mg once daily) is expected to provide new standards for secondary stroke prevention [64].

**Table 2:** Included studies on treatment of intracranial atherosclerosis [63, 64]

Studies/ Trials	Study duration	Type of study	Degree of stenosis	Interventions	Population	Outcome	Event rates
Comparison of Warfarin versus Aspirin for symptomatic ICAS (WASID) [12]	1999–2003	Randomized-Controlled Clinical Trial	50-99%	Aspirin 650 mg twice a day versus Warfarin (target INR:2–3)	569	Warfarin non superior for stroke prevention and with a higher rate of major hemorrhages and all-caused death compared to aspirin	Death: 4.3% in the aspirin group versus 9.7% in the warfarin group; major hemorrhage: 3.2% versus 8.3%, respectively; myocardial infarction or sudden death: 2.9% versus 7.3%, respectively
Comparison of stenting versus aggressive medical management for severe ICAS (SAMMPRIS) [70]	2008–2011 (enrollment was stopped because of safety concerns regarding the risk of periprocedural stroke or death in the PTAS group)	Randomized-Controlled Clinical Trial	70-99%	Aspirin 325 mg per day combined with clopidogrel 75 mg per day for 90 days and management of risk factors (blood pressure, low density lipoprotein cholesterol, diabetes, smoking, obesity, exercise) versus percutaneous transluminal angioplasty and stenting (PTAS) with the use of Wingspan stent system	451	Aggressive medical management (combination antiplatelet therapy and intensive management of risk factors) is superior to PTAS with the use of the Wingspan stent system	30-day rate of stroke or death was 14.7% in the PTAS group (nonfatal stroke: 12.5%; fatal stroke: 2.2%) versus 5.8% in the medical-management group (nonfatal stroke: 5.3%; non-stroke-related death: 0.4%)
Comparison of clopidogrel plus aspirin versus aspirin alone for reducing embolization in patients with acute symptomatic cerebral or carotid artery stenosis (CLAIR study) [88]	2003–2008	Randomized-Controlled Clinical Trial	50-99%	Dual therapy of clopidogrel 300 mg for the first day and then 75 mg daily plus 75–160 mg aspirin daily for 7 days versus monotherapy 75–160 mg aspirin once daily for 7 days	100	Combination therapy with clopidogrel and aspirin is more effective than aspirin alone in reducing microembolic signals in patients with predominantly intracranial symptomatic stenosis	On day 2, 14 of 45 patients in the dual therapy group and 27 of 50 in the monotherapy group had microembolic signals. At day 7, ten of 43 patients in the dual therapy group and 26 of 51 in the monotherapy group had microembolic signals. The number of adverse events was similar between the two groups.
Comparison of balloon-expandable stent plus medical therapy versus medical therapy alone; the Vitesse Intracranial Stent Study for Ischemic Stroke Therapy (VISSIT) trial [71]	2009–2012	Randomized-Controlled Clinical Trial	70-99%	Medical therapy consisted of clopidogrel (75 mg daily) for the first 3 months after enrollment plus aspirin (81–325 mg daily) for the study duration, and management of risk factors compared to balloon-expandable stent placement	112	Among patients with symptomatic ICAS, the use of a balloon-expandable stent compared with medical therapy resulted in an increased 12-month risk of added stroke or TIA in the same territory, and increased 30-day risk of any stroke or TIA	Intracranial hemorrhage within 30 days occurred in 8.6% of the patients in the stenting group versus none in the medical group, the 1-year primary outcome of stroke or hard TIA occurred in 36.2% of the patients in the stenting group versus 15.1% of the patients in the medical group; worsening of baseline disability score (modified Rankin Scale) occurred in 24.1% of the patients in the stent group versus 11.3% of the patients in the medical group.
Comparison of stenting versus medical treatment in patients with ICAS; the Vertebral Artery Stenting Trial (VAST) [72]	2008–2013	Randomized-Controlled Clinical Trial	50-99%	Patients randomized to stenting received clopidogrel 75 mg daily starting at least five days before the procedure and continued for 30 days after the procedure. Patients not on clopidogrel the day before the procedure were loaded with 300 mg clopidogrel at least six hours before stenting	115	Stenting of symptomatic vertebral artery stenosis did not lower the risk of stroke and presented more adverse events	Three patients in the stenting group had vascular death, myocardial infarction, or any stroke within 30 days after the start of treatment (5%) versus one patient in the medical treatment group (2%). During a median follow-up of 3 years, seven (12%) patients in the stenting group and four (7%) in the medical treatment group had a stroke in the territory of the symptomatic vertebral artery; 11 (19%) patients in the stenting group and ten (17%) in the medical treatment group had vascular death, myocardial infarction, or any stroke.
Αssessment of safety and effectiveness for percutaneous transluminal angioplasty and stenting (PTAS) in patients with symptomatic ICAS; systematic review and meta-analysis [73]	2016	Meta-analysis	50-99%	Meta-analysis of three studies: SAMMPRIS, VAST and VISSIT	678	PTAS is associated with adverse early and long-term outcomes and should not be recommended in patients with symptomatic ICAS	PTAS was associated with a higher risk of recurrent ischemic stroke in the territory of qualifying artery both within 30 days [risk ratio (RR) = 2.21] and 1 year (RR = 1.92). PTAS was also related to a higher risk of any ischemic stroke within 30 days from the index event (RR = 2.08). The risk for intracranial hemorrhage was found to be higher in PTAS patients both within 30 days (RR = 10.60) and 1 year (RR = 8.15). The composite outcome of any stroke or death within 1 year (RR = 2.29) and 2 years (RR = 1.52) was higher in PTAS than in medical therapy. PTAS was associated with a higher risk of any stroke or death within 2 years in the symptomatic ICAS subgroup located in posterior circulation (RR = 2.37)
The Wingspan Stent System Post Market Surveillance (WEAVE) trial assessed the periprocedural safety of the Wingspan Stent system in the treatment of symptomatic patients with ICAS and specific criteria [75]	2012–2018	Postmarketing Surveillance Trial	70-99%	Patients aged 22 to 80 years, with symptomatic ICAS of 70–99%, baseline modified Rankin Scale score ≤ 3, ≥2 strokes in the vascular territory of the stenotic lesion with at least 1 stroke while on medical therapy, and stenting of the lesion ≥ 8 days after the last stroke, underwent angioplasty and stenting with the Wingspan stent	152	With experienced interventionalists, and proper patient selection following the on-label usage guidelines, the use of the Wingspan stent for ICAS demonstrated a low periprocedural complication rate and excellent safety profile	A total of 97.4% (148/152) patients were event-free at 72 h, 1.3% (2/152) had nonfatal strokes, and 1.3% (2/152) of patients died.
Comparison of Drug-Eluting Stent (DES) With Bare-Metal Stent (BMS) in patients with symptomatic high-grade ICAS [79]	2015–2018	Randomized-Controlled Wingspan Clinical Trial	70-99%	Patients were randomly assigned to receive DES (NOVA intracranial sirolimus-eluting stent system) or BMS (Apollo intracranial stent system) treatment in a 1:1 ratio	263	Compared with BMS, DES reduced the risks of in-stent restenosis and ischemic stroke recurrence in patients with symptomatic high-grade ICAS	The 1-year in-stent restenosis rate was lower in the DES group than in the BMS group (10 [9.5%] versus 32 [30.2%]). The DES group also had a significantly lower ischemic stroke recurrence rate from day 31 to 1 year (1 [0.8%] versus 9 [6.9%]). No significant difference in the rate of any stroke or death within 30 days was observed between the DES and BMS groups (10 [7.6%] versus 7 [5.3%]).
Comparison of stenting plus medical therapy versus medical therapy alone in patients with symptomatic severe ICAS; the China Angioplasty and Stenting for Symptomatic Intracranial Severe Stenosis (CASSISS) trial [74]	2014–2019	Randomized-Controlled Clinical Trial	70-99%	Medical therapy (dual antiplatelet therapy with aspirin 100 mg and clopidogrel 75 mg daily for 90 days followed by aspirin or clopidogrel alone daily, and stroke risk factor control) plus stenting compared to medical therapy alone	358	Among patients with transient ischemic attack or ischemic stroke due to symptomatic severe ICAS, the addition of percutaneous transluminal angioplasty and stenting to medical therapy, compared with medical therapy alone, resulted in no significant difference in the risk of stroke or death within 30 days or stroke in the qualifying artery territory beyond 30 days through 1 year	For the stenting plus medical therapy group versus medical therapy alone, no significant difference was found for the primary outcome of risk of stroke or death (8.0% [14/176] versus 7.2% [13/181]). Of the 5 prespecified secondary end points, none showed a significant difference including stroke in the qualifying artery territory at 2 years (9.9% [17/171] versus 9.0% [16/178]) and 3 years (11.3% [19/168] versus 11.2% [19/170]). Mortality at 3 years was 4.4% (7/160) in the stenting plus medical therapy group versus 1.3% (2/159) in the medical therapy alone group.
Comparison of extracranial-intracranial arterial bypass plus medical treatment versus medical treatment alone, in patients with atherosclerotic narrowing or occlusion of the ipsilateral internal carotid or middle cerebral artery; the International Cooperative Study of Extracranial/Intracranial Arterial Anastomosis (EC/IC Bypass study) [82]	1977–1982	Randomized-Controlled Clinical Trial	Narrowing or occlusion of the ipsilateral internal carotid or middle cerebral artery	714 patients were randomly assigned to the best medical care (acetylsalicylic acid 325 mg four times a day), and 663 to the same regimen with the addition of bypass surgery joining the superficial temporal artery and the middle cerebral artery	1377	Extracranial-intracranial anastomosis is not effective in preventing cerebral ischemia in patients ICAD in the carotid and middle cerebral arteries	30-day surgical mortality and major stroke morbidity rates were 0.6% and 2.5%, respectively. Nonfatal and fatal stroke occurred both more frequently and earlier in the patients operated on. Secondary survival analyses comparing the two groups for major strokes and all deaths, for all strokes and all deaths, and for ipsilateral ischemic strokes demonstrated a similar lack of benefit from surgery. Separate analyses in patients with different angiographic lesions did not identify a subgroup with any benefit from surgery.

Abbreviations:

BMS: Bare metal stent; CASSISS: China Angioplasty and Stenting for Symptomatic Intracranial Severe Stenosis; CLAIR: Clopidogrel Plus Aspirin for Infarction Reduction; DES: Drug eluting stent; EC/IC Bypass study: International Cooperative Study of Extracranial/Intracranial Arterial Anastomosis; ICAS: Intracranial Atherosclerotic Stenosis; PTAS: Percutaneous Transluminal Angioplasty and Stenting; SAMMPRIS: Stenting and Aggressive Medical Therapy for Preventing Recurrent Stroke in Intracranial Stenosis; VAST: Vertebral Artery Stenosis Trial; VISSIT: Vitesse Intracranial Stent Study for Ischemic Stroke Therapy; WASID: Warfarin-Aspirin Symptomatic Intracranial Disease; WEAVE: Wingspan Stent System Post Market Surveillance

Management of cardiovascular risk factors, including hypertension, diabetes and hyperlipidemia, has proven effective for secondary AIS prevention in the presence of ICAS. Subgroup analyses of the WASID and the SAMMPRIS trials demonstrated significantly lower rates of stroke and TIA recurrence in patients with ICAS maintaining a mean systolic/diastolic blood pressure below 140/90 mmHg (130/80 mmHg if diabetic), LDL cholesterol below 70 mg/dl, and glycosylated hemoglobin (HbA1c) levels below 7% [65]. High-intensity statins (i.e., atorvastatin or rosuvastatin) comprise first-line treatments for lowering LDL levels in patients with ICAS, with intensive statin therapy correlated with time-dependent plaque stabilizing effects [66, 67]. When aggressive LDL targets (< 55 mg/dL) for high-risk patients with history of TIA or stroke cannot be attained with statin therapy alone, the addition of ezetimibe and/or PCSK9 (proprotein convertase subtilisin/kexin type 9) inhibitors is recommended [21, 63]. In addition, lifestyle interventions such as smoking cessation, weight loss, dietary modification and regular physical activity are recommended for secondary stroke prevention [63].

Beyond the current mainstay of pharmacological therapies, novel treatments for ICAS management are underway. These comprise agents that target Lp(a) and novel antidiabetics. Elevated Lp(a) levels, especially above 50 mg/dl, are associated with an increased risk of ICAS. Notably, currently available lipid-lowering drugs are significantly limited in attenuating serum Lp(a) levels. RNA-based drugs like pelacarsen, olpasiran, SLN360, and LY3819469 are currently under investigation, with RCTs expected to soon provide evidence on their clinical safety and efficacy in lowering Lp(a) and their potential role in ICAS management [68]. Additionally, glucagon-like-peptide-1 receptor agonists (GLP-1 RAs) and the novel dual (glucose-dependent insulinotropic peptide) GIP/GLP-1 RA tirzepatide have been shown to attenuate the risk of ischemic stroke, while several studies are currently underway to unfold the full neuroprotective potential of these novel agents [69]. Furthermore, bempedoic acid is another novel lipid-lowering drug that can be used in addition to high-dose statins or in combination with ezetimibe to reach LDL-c targets, although there are insufficient data on its effects on reducing fatal and nonfatal strokes [65].

### Endovascular and surgical treatment

Despite optimal medical management, symptomatic ICAS poses a significant risk of stroke recurrence and mortality. Three seminal RCTs, the SAMMPRIS, VISSIT (Vitesse Intracranial Stent Study for Ischemic Stroke Therapy) and VAST (Vertebral Artery Stenosis Trial) investigated the safety and efficacy of angioplasty and stenting in patients with symptomatic ICAS (Table 2) [70–72]. These trials enrolled patients with moderate and severe ICAS who had experienced AIS or TIA attributed to ICAS within 30 days prior to enrollment. Both SAMMPRIS and VISSIT were terminated prematurely due to higher-than-expected periprocedural risks in the stenting groups and much lower-than-expected rates of ischemic stroke among participants receiving medical treatment alone, while VAST demonstrated lack of benefit regarding stroke reduction and a higher incidence of adverse events in the stenting group. In the SAMMPRIS trial, the early benefit of aggressive medical management over stenting for patients with high-grade ICAS at the 30th day follow-up persisted in the extended follow-up. In particular, the occurrence of primary endpoint events (i.e., recurrent stroke, any major intracranial or systemic hemorrhage, or death) differed significantly between the medical versus stenting group (5.8% versus 14.7%) at day 30. Accordingly, recurrence rates of the primary endpoint events were 12.6% versus 19.7% in the medical versus stenting group at year 1, and 14.1% versus 20.6% at 2 years [62]. A previous meta-analysis by our group including data from the SAMMPRIS, VISSIT and VAST trials, demonstrated that percutaneous transluminal angioplasty and stenting (PTAS) was associated with higher risk of AIS, intracranial hemorrhage and death compared to medical treatment alone (Table 2) [73]. The recent China Angioplasty and Stenting for Symptomatic Intracranial Severe Stenosis (CASSISS) trial, which compared stenting plus medical therapy versus medical therapy alone in highly-selected patients with symptomatic severe ICAS at high-volume enrolling sites, concluded that the addition of PTAS to medical therapy, did not result in a significant difference in the risk of stroke or death within one month, compared with medical therapy alone (Table 2) [74]. Accordingly, current AHA/ASA and ESO guidelines advise against stenting as the initial treatment in unselected patients with symptomatic ICAS [21, 63].

Regarding the potential benefit of endovascular therapy in selected patients, the WEAVE (Wingspan Stent System Post Market Surveillance) trial supported the use of the Wingspan stent in a subgroup of symptomatic ICAS patients who do not respond to aggressive medical treatment (Table 2) [75]. These include patients aged 22 to 80 years, with symptomatic ICAS of 70–99%, baseline modified Rankin Scale score ≤ 3, ≥2 strokes in the vascular territory of the stenotic lesion with at least 1 stroke while on medical therapy, and stenting of the lesion ≥ 8 days after the last stroke. This trial concluded that with experienced interventionalists and proper patient selection, the use of the Wingspan stent is associated with low periprocedural complication rates. As a result, ESO guidelines suggest considering endovascular treatment with angioplasty and/or stenting as rescue therapy in selected patients with symptomatic high-grade ICAS after AIS recurrence despite best medical treatment [21].

There is currently ongoing research that aims to identify high-risk subgroups, such as those exhibiting hemodynamic impairment, that could potentially benefit from revascularization. The VERITAS (Vertebrobasilar Flow Evaluation and Risk of Transient Ischemic Attack and Stroke Study) trial, employing Quantitative MRA, demonstrated that patients with reduced flow distal to posterior circulation stenosis or occlusion faced a substantially heightened risk of future stroke compared to those with normal flow, therefore suggesting that high-risk, distal flow-compromised patients may be a candidate population for future trials of endovascular intervention [76]. Another study investigated hemodynamic markers (border-zone infarct pattern and impaired collateral flow) in the anterior circulation of symptomatic patients with ICAS. This was a post-hoc analysis of patient-data from the medical treatment arm of the SAMMPRIS trial concluding that the risk of stroke recurrence was higher in those with border-zone infarct pattern, suggesting that stenting may be more effective than medical management in the setting of hemodynamic insufficiency [77].

In the same context, novel strategies for angioplasty and stenting are currently under investigation A new generation of balloon-expanding stents and new PTA (percutaneous transluminal angioplasty)-balloons that can be used as microcatheters for stent implantation may provide improved technical results in symptomatic patients with ICAS [21]. The slender profile of angioplasty balloons in contrast to stent-bearing catheters may result in less ostial occlusion of perforators induced by plaque “snow-plowing”, which entails the forced displacement of atheromatous debris into perforator origins. Submaximal angioplasty, characterized by the gradual expansion of a smaller balloon to 50–80% of the normal vessel diameter, is also under investigation as alternative strategy for managing ICAS [78]. Although there is some evidence in the literature that drug-eluting stents may reduce the risk of in-stent restenosis and AIS recurrence compared to bare-metal stents in patients with symptomatic high-grade ICAS, further large-scale RCTs are needed to provide robust evidence on the safety and efficacy of these novel techniques (Table 2) [79].

There is emerging expert opinion evidence indicating that acute stenting, as part of complex thrombectomy procedures in AIS patients with large vessel occlusion (LVO) due to underlying ICAD, may yield improved clinical outcomes compared to patients who do not undergo stenting [80]. A recent meta-analysis of four studies, including patients with failed mechanical thrombectomy, revealed that those who received stent rescue had significantly higher rates of favorable clinical outcomes and lower mortality rates at 90 days compared to non-stented patients, without an increased risk of symptomatic intracerebral hemorrhage [81]. However, based on current ESO guidelines, there is no clear indication for performing stenting in patients undergoing mechanical thrombectomy for AIS due to ICAD as standard clinical practice, nor for the infusion of glycoprotein IIb/IIIa inhibitors after initial mechanical thrombectomy [21].

Concerning surgical treatment, bypass surgery has been explored as an alternative procedure for managing ICAS, although its clinical utility in non-Moyamoya ICAS remains limited. The EC/IC Bypass study (International Cooperative Study of Extracranial/Intracranial Arterial Anastomosis) failed to demonstrate the efficacy of extracranial to intracranial anastomosis to effectively prevent cerebral ischemia in ICAS (Table 2) [64, 82]. Evidence from earlier studies has limited the use of bypass surgery in clinical practice. Nevertheless, in patients with severe ICAS and recurrent ischemic events or hemodynamic failure despite intensive medical therapy, it has been reported that STA (superficial temporal artery)-MCA (middle cerebral artery) bypass surgery performed in specialized neurovascular centers has an acceptable perioperative risk of 4.3% and should arguably be reserved as a last-tier treatment option for carefully selected patients [83]. Notwithstanding the previous evidence, the current ESO guidelines recommend against neurosurgical procedures in patients with AIS or TIA related to high grade ICAS [21]. Encephaloduroarteriosynangiosis (EDAS) represents another indirect revascularization technique. In this approach, donor arteries, such as the superficial temporal artery and middle meningeal arteries, are positioned proximal to the dural and cortical arteries and distal to the intracranial stenosis. Over time, this facilitates the development of a collateral network between the donor artery and the nearby superficial brain vessels, obviating the need for surgical anastomosis. In a recent study, thirteen patients with symptomatic ICAS not suitable for angioplasty or stenting underwent EDAS over a 9-year period, with the results suggesting that EDAS is a safe and effective method to improve cerebral blood flow in the presence of ICAS [84].

As evidenced from the above, limited data exist for managing patients with stenoses less than 50% (i.e., ICAD). Given that many AIS and TIA result from non-stenotic unstable atherosclerotic plaques, high-dose statin therapy for plaque stabilization may be as effective in patients with ICAD without ICAS [44, 67]. Additionally, antiplatelets such as aspirin seem applicable in ESUS cases attributed to non-stenotic atherosclerotic plaques [30]. There is also first promising evidence that anti-inflammatory agents, including colchicine, may reduce the risk of AIS and TIA recurrence in patients with non-cardioembolic stroke and thus may hold potential for future use in ICAD management [85, 86]. Clearly, substantial progress remains to be made in the management of ICAD. Beyond the currently available medical and interventional procedures, future research aims to repurpose or develop new antithrombotic regimens (e.g., direct thrombin inhibitors, factor XI inhibitors), promote anti-inflammatory therapeutic strategies and refine endovascular treatments (e.g., submaximal balloon angioplasty), and explore novel strategies such as indirect bypass and ischemic preconditioning.

In summary, to date, RCTs have predominantly addressed symptomatic ICAS management, leaving a notable gap in evidence for patients with non-stenotic ICAD. Current medical management centers on antithrombotic therapy, with antiplatelet therapy emerging as a safer option compared to anticoagulants in reducing stroke recurrence among patients with ICAS. Aggressive control of cardiovascular risk factors, including hypertension and hyperlipidemia, alongside high-intensity statin therapy, remains pivotal for secondary stroke prevention. While endovascular therapies, including angioplasty and stenting, have been explored, recent trials emphasize their limited benefit over optimal medical therapy alone, except in selected high-risk cases. Ongoing research explores novel pharmacological and interventional strategies to optimize ICAS and ICAD management, aiming to effectively reduce stroke risk.

## Conclusions

The current comprehensive analysis of the latest evidence on ICAD, distinct from ICAS, aimed to highlight the evolving epidemiological landscape of intracranial atherosclerosis. Genetic, environmental, and epigenetic factors confer increased susceptibility to ICAD among certain ethnicities and races. However, emerging evidence indicates a paradigm shift in clinical practice, with a marked increase in the detection rates of ICAD among White populations. ICAD is currently estimated to affect up to 1 out of every 2 White patients presenting with AIS or stroke of undetermined etiology. These estimates underscore the previously undetected burden of intracranial atherosclerosis and the urgent need for a comprehensive screening.

Diagnosing non-stenotic ICAD presents significant clinical challenges that necessitate a multimodal approach beyond the traditional imaging techniques primarily focused on ICAS. HRVW-MRI is an indispensable tool, particularly for patients presenting with cryptogenic stroke or ESUS. Furthermore, the epidemiological insights suggesting a more uniform distribution of ICAD across diverse racial and ethnic groups necessitate a reassessment of risk factors and screening protocols. While the conventional management of ICAS includes pharmacotherapy, risk factor modification, and endovascular therapy, substantial progress remains to be made in the management of ICAD. Future research aims to identify novel therapeutic strategies and refine endovascular treatments for selected high-risk patients. With a growing understanding of ICAD, a multifaceted approach that prioritizes prevention, early detection, and targeted treatment is warranted. This approach is crucial for improving patient outcomes and addressing the hidden burden of intracranial atherosclerosis globally.

## Data Availability

All data analyzed in the present study have been included in the present article.

## Abbreviations

AHA: American Heart Association
AIS: Acute ischemic stroke
ASA: American Stroke Association
CFD: Computational fluid dynamics
CTA: Computed tomogrpahy angiography
DSA: Digital Subtraction Angiography
ESO: European Stroke Organization
ESUS: Embolic stroke of undetermined source
GIP: Glucose-dependent insulinotropic peptide
GLP-1: Glucagon-like-peptide-1
GLP-1 RA: Glucagon-like-peptide-1 receptor agonists
HR-MRA: High resolution magnetic resonance angiography
HR-MRI: High resolution magnetic resonance imaging
HRVW-MRI: High resolution vessel wall magnetic resonance imaging
ICAD: Intracranial atherosclerotic disease
ICAS: Intracranial atherosclerotic stenosis
LDL: Low-density lipoprotein
LVO: Large vessel occlusion
MES: Microembolic signals
MRA: Magnetic resonance angiography
NOACs: Novel Oral Anticoagulants
PETRA-MRA: Pointwise encoding time reduction with radial acquisition magnetic resonance angiogrpahy
PTAS: Percutaneous transluminal angioplasty and stenting
RCTs: Randomized Controlled Trials
SMC: Smooth muscle cell
TCD: Transcranial doppler ultrasound
TIA: Transient ischemic attack
TOF-MRA: Time-of-flight magnetic resonance angiography
VMR: Vasomotor reactivity
